# Oral Microbiota during Childhood and Its Role in Chemotherapy-Induced Oral Mucositis in Children with Cancer

**DOI:** 10.3390/pathogens11040448

**Published:** 2022-04-07

**Authors:** Silvia Triarico, Pierpaolo Agresti, Emanuele Rinninella, Maria Cristina Mele, Alberto Romano, Giorgio Attinà, Palma Maurizi, Stefano Mastrangelo, Antonio Ruggiero

**Affiliations:** 1 UOSD di Oncologia Pediatrica, Dipartimento di Scienze della Salute della Donna, del Bambino e di Sanità Pubblica, Fondazione Policlinico Universitario A. Gemelli IRCCS, Argo A. Gemelli 8, 00168 Rome, Italy; silvia.triarico@guest.policlinicogemelli.it (S.T.); alberto.romano@guest.policlinicogemelli.it (A.R.); giorgio.attina@policlinicogemelli.it (G.A.); palma.maurizi@unicatt.it (P.M.); stefano.mastrangelo@unicatt.it (S.M.); 2 Scuola di Specializzazione in Pediatria, Università Cattolica del Sacro Cuore, Largo F.sco Vito 1, 00168 Rome, Italy; piero.agresti@gmail.com; 3 UOC di Nutrizione Clinica, Dipartimento di Scienze Mediche e Chirurgiche, Fondazione Policlinico Universitario A. Gemelli IRCCS, 00168 Rome, Italy; emanuele.rinninella@unicatt.it (E.R.); mariacristina.mele@unicatt.it (M.C.M.); 4 Dipartimento di Medicina e Chirurgia Traslazionale, Università Cattolica del Sacro Cuore, Largo F.sco Vito 1, 00168 Rome, Italy; 5 Dipartimento di Scienze della Vita e Sanità Pubblica, Università Cattolica del Sacro Cuore, Largo F.sco Vito 1, 00168 Rome, Italy

**Keywords:** oral microbiota, children, cancer, chemotherapy, oral mucositis

## Abstract

The human oral cavity harbors the second most abundant microbiota after the gastrointestinal tract, with over 700 species currently identified in the oral microflora. The oral microbiota develops from intrauterine life and after birth is continuously shaped by several influencing factors. The perturbation of the diversity and proportions of species within the oral microbiota leads to dysbiosis and associated increased risk of local and systemic diseases. In children who receive chemotherapy for cancer, oral mucositis is a common and painful side effect that decreases quality of life (QoL) and treatment adherence. The oral microbiota undergoes a substantial dysbiosis as an effect of cancer and its treatment, characterized by lower richness and less diversity. Furthermore, this dysbiosis seems to promote pro-inflammatory cytokine release and pro-apoptotic mediators, enhancing the oral tissue damage. Further studies on the role of the oral microbiota in the pathogenesis of oral mucositis should be performed among children with cancer who receive chemotherapy, to find preventive and protective factors against the pathogenesis of oral mucositis.

## 1. Introduction

Our human body is not an autonomous organism, but consists of many resident microorganisms that coevolve and coexist with our cells in a symbiotic relationship. This complex, dynamic end heterogeneous ecosystem is fundamental to our health and physiology [1]. Together with our symbiotic microbial residents, we form a “holobiont” organism. The Nobel Prize Joshua Lederberg first called the ecological community of symbiotic, commensal and pathogenic microbial residents in our body the “microbiome” in 2001. “Microbiome” refers to the collection of genomes from all the microorganisms in the environment, whereas “microbiota” usually indicates microorganisms that are found within a specific environment [2]. The microbiome transforms in parallel with host metabolic, immunologic and cognitive development, contributing to host normal physiologic functions. Moreover, pathological changes in the host can affect the balance of the microbial species within these communities [3]. The Human Microbiome Project (HMP) has been carried out to understand the relationship that links the microbiome and the host health-related outcomes [4].

The human oral cavity is a major gateway to the human body, harboring the second most abundant microbiota after the gastrointestinal tract. The oral microbiota is constituted by the microorganisms found in the human oral cavity (that includes teeth, gingival sulcus, attached gingiva, tongue, cheek, lip, hard and soft palate) and its contiguous extensions (such as tonsils, pharynx, esophagus, Eustachian tube, middle ear, trachea, lungs, nasal passages and sinuses), until the distal esophagus [5]. In 1674, Antony van Leeuwenhoek first discovered both protists and bacteria, observing his dental plaque using a microscope. Numerous later studies isolated in culture and named about 280 bacterial species from the oral cavity [6]. Currently, over 700 species have been identified in the oral microflora, using 16S ribosomal RNA (rRNA) sequencing from DNA salivary samples and the Operational Taxonomic Units (OTUs), which refer to clusters of organisms grouped by DNA sequence similarity of a specific taxonomic marker gene [7,8,9]. Furthermore, the Human Oral Microbiome Database (HOMD) provides information on bacterial communities present in the oral cavity, pharynx, nasal passages, sinuses, and esophagus [10].

The 16S rDNA profiling categorized the bacteria into six major phyla that constitute about 96% of total oral bacteria in a healthy subject: *Firmicutes, Actinobacteria, Proteobacteria, Fusobacteria, Bacteroidetes and Spirochaetes*. The other major constituents of oral microbiota include *Actinomyces, Atopobium, Corynebacterium, Rothia, Bergeyella, Capnocytophaga, Prevotella, Granulicatella, Streptococcus, Veillonella, Campylobacter, Cardiobacterium, Haemophilus, Neisseria, TM7* [6,11,12].

The oral microbiota is crucial for keeping homeostasis in the oral cavity. The perturbation of the diversity and proportions of species within the microbiota, with a single or few species predomination, leads to dysbiosis and associated increased risk of local and systemic diseases [13].

Childhood is a chief time for the founding of the adult oral microbiota. In fact, the shaping of an individual’s oral microbiota may be influenced by several factors (genetic determinants, pregnancy term, delivery mode, and feeding method) that occur in early childhood. Furthermore, specific changes occur in oral microbiota diversity in several healthy pediatric conditions (early childhood caries, pediatric obesity, celiac disease, autism, Henoch-Shonlein purpura disease, pediatric appendicitis, pediatric inflammatory bowel diseases, pediatric hemato-oncological diseases), as described in various cross-sectional and case-control studies [14]. The analysis of the oral microbiota in various clinical conditions allows one to draw a specific disease risk profile for each patient and to set treatment options as personalized as possible [15,16]. Pediatric patients with cancer are a peculiar set of patients, in which the study of the oral microbiota may provide important information about the prognosis and the most common illness-related complications, such as chemotherapy-induced mucositis [17].

In this review, we describe the main aspects of the physiological development of the oral microbiota during childhood and its pathological changes during pediatric chronic diseases. Furthermore, we focus on the effects of chemotherapy on the composition of the oral microbiota in pediatric patients with cancer. We investigate the possible relationship between the alterations of the oral microbiota and the onset of oral mucositis, in order to find preventive and protective factors against the pathogenesis of oral mucositis in this peculiar setting of patients.

## 2. Physiological and Pathological Changes of the Oral Microbiota during Childhood

The colonization of the oral cavity by microorganisms takes place from intrauterine life (through amniotic fluid, uterine membranes and meconium) and after birth it is never stable, but continuously shaped by several influencing factors, such as pregnancy term, delivery mode, and feeding method [18,19,20].

Premature newborns may be hospitalized in a neonatal intensive care unit (NICU) for a variable time, thus they may be colonized by environmental microbes, including commensal and pathogenic microorganisms, such as *Streptococcus, Staphylococcus, Neisseria* and *Enterobacteriaceae* (usually found on continuous positive airway pressure machines, stethoscopes, ventilators, incubators, radiant warmers), *Geobacillus, Halomonas, Shewanella, Acinetobacter*, and *Gemella* (discovered on computer screens, a computer mouse, freezer handles, hand sanitizer bottles, door handles, telephones, heart rate monitor screens, alarm buttons) [21]. Cortez et al. demonstrated that the oropharyngeal administration of colostrum (OAC) in premature neonates (that may be unable to feed with their mother’s milk) may stimulate early maturation of the oral cavity, with increased abundance of beneficial bacteria, such as *Staphylococcus, Bifidobacterium* and *Bacteroides* [22].

The type of delivery (vaginal delivery or cesarean section) seems to influence the composition of the oral microbiota only in the first week after birth. Newborns born from vaginal delivery have early colonization by bacteria present in the vaginal canal, such as *Lactobacilli, Prevotella, Bacterioides*, while those born by caesarean section show early colonization by *Veillonella, Corynebacterium, Propionibacterium* and *Staphylococci*. However, in both cases, a preponderance of Streptococci may be observed with the initiation of breastfeeding [18].

During the lactation period, there is observed a predominance of Gram-positive bacteria, like *Streptococcus, Granulicatella* and *Veillonella*. *Streptococci* are especially present in breast milk and they are very able to adhere to mucous membranes [23].

Then the main changes in the oral microbiota occur between 6 and 24 months [24], with the consequent transition from a predominance of *Streptococcus mutans, Fusobacteria, Tenericutes, TM7,* and *SR1*. With the appearance of primary dentition, specific niches colonized by different bacterial species are observed; furthermore, oral bacteria diversity continues to grow through time, with about 550 OTUs at the end of primary dentition [23]. With the appearance of the definitive dentition, the oral microbiota of each child can be defined as personalized. The oral microbiota of adults is more influenced by oral hygiene habits, while in children of the same familiar nucleus, oral hygiene seems to not affect the diversity of bacterial species [25].

Among children, a significant difference in bacterial species of the oral cavity is related to weight and lifestyle. Burcham et al. found that obese children and those with a BMI near to obesity have an oral microbiota poorer in bacterial species than non-obese children [25].

Another study analyzed the difference in oral microbiota between children with a sedentary lifestyle and those with an active lifestyle, finding low bacterial richness (with a difference of about 12 OTUs) in sedentary children, with consequent adverse metabolic markers, such as adiposity and insulin resistance. Moreover, overweight children tend to have a predominance of species like *Streptococcus* and *Veillonella*, without competition from other bacterial species, with a consequent greater preponderance of oral cavity pathologies, such as caries and periodontitis [26].

A lot of studies have already analyzed the dysbiosis of the gut microbiota especially in the study of inflammatory bowel disease (IBD), celiac disease and other systemic diseases [27,28].

More recently the relationship between the microenvironment of the oral cavity, oral pathologies and systemic diseases have been widely analyzed. The proposed mechanisms of these oral-systemic links include the spread of bacteria from the oral cavity in the form of bacteremia or circulating bacterial toxins, which may trigger an increase of circulating pro-inflammatory cytokines and a weakness of the immune system, favoring systemic diseases or their complications [29,30,31].

Dental caries is a chronic illness very common among children, strongly correlated with certain species of oral bacteria. Streptococcus mutans, an acid-producing bacteria, is very involved and associated with the pathogenesis of caries [32], which originate from the removal of the biofilm present on the surface of the teeth. Moreover, elevated levels of *S. salivarius, S. sobrinus*, and *S. parasanguinis* are also associated with caries, especially in subjects with absent or low levels of S. mutans, suggesting these species as alternative pathogens for oral caries. *Veillonella* has been more associated with an increased risk of future caries among children without a previous history of caries, other than S. mutans or other acid-producing species [33].

The oral microbiota has been investigated in children with systemic diseases, such as celiac disease, Henoch-Schonlein purpura and Crohn’s disease. The salivary microbiota of children with celiac disease is different from healthy controls. Coeliac children have very little prevalence of salivary *S. mutans, Lactobacilli,* and *Anaerobes*, but an increased level of *Enterobacteriaceae.* Consistently, 16S rRNA gene-based meta-genetic data showed very low values of richness estimator (Chao1) and diversity index (Shannon) in the saliva of celiac children [34].

Children with Henoch-Schonlein purpura (HSP) exhibit a higher oral microbial diversity and richness than their healthy controls, with a predominance of *Firmicutes, Proteobacteria* and *Bacteroidetes* [35].

In Crohn’s disease, oral lesions are pathognomonic and sometimes precede the onset of intestinal symptoms. The analysis of the oral microbiota showed a higher presence of *Fusobacteria* and *Firmicutes* than their healthy counterparts [36].

The association between dysbiosis and the onset of neoplasms was already investigated in oncologic patients. In patients with cancer, the microbial host homeostasis may be disrupted by cancer itself, by the bactericidal effect of chemotherapy and antibacterial agents adopted against secondary infections, but also by the immunodeficiency induced by chemotherapy [37].

Although a direct causality has not yet been proven, individual members of the oral microbiome may promote tumorigenesis and may be adopted as potential biomarkers in several types of adult cancer. As examples of specific organisms as biomarkers, an increase in *Fusobacterium nucleatum* has been linked to colorectal cancer, *Porphyromonas gingivalis* and *Fusobacterium* spp. have been linked to pancreatic cancer [38].

Furthermore, in some individuals the presence of chronic periodontitis and chronic oral colonization by *Porphyromonas gingivalis, Fusobacterium nucleatum* and *Candida* can induce cellular changes consistent with dysplasia that may lead to oral carcinoma, and therefore requires treatment [39].

## 3. Oral microbiota and Chemotherapy-Induced Oral Mucositis in Children

Between 2015 and 2030, 6.7 million cases of childhood cancer are estimated worldwide, with more than 450,000 cases per year [40]. According to the NCI Surveillance, Epidemiology, and End Results (SEER) Program, each year from 2014 to 2018 there were 17.8 cancer diagnoses per 100,000 children aged younger than 15 years and 77.4 cancer diagnoses per 100,000 adolescents and young adults aged 15 to 39 years. Leukemias, brain tumors and lymphomas represent the largest diagnostic groups among the under 15-year-olds. The most frequent single diagnoses are acute lymphoblastic leukemia, astrocytoma, neuroblastoma, non-Hodgkin lymphoma, and nephroblastoma [41]. The cumulative overall survival of pediatric cancers has increased during the last years, with more than 84% of children diagnosed with cancer expected to survive at least 5 years, because of the adoption of international cooperative treatment regimens and the growing attention to supportive care [40].

Common side effects associated with the treatment of pediatric cancers are oral mucositis, which may produce pain, difficulty feeding, malnutrition, prolonged hospitalization and potential bloodstream infection, inducing a significant decline in patients’ quality of life (QoL) and compliance to the treatment [42,43].

The incidence rate of oral mucositis ranges from 52% in patients who receive standard chemotherapy to 100% in patients who receive high-dose chemotherapy. If not managed with adequate measures, oral mucositis represents an important limiting factor of chemotherapy and may worsen patient prognosis and compliance [44]. Malnutrition, pre-existing medical conditions, alterations in salivary production and composition, and poor oral health have been reported as patient-associated risk factors for the development of oral mucositis [45,46].

Saliva is the first barrier for defending against microbial invasion through mechanical, nonimmunologic and immunologic functions. Its continuous flow eliminates food debris and exogenous harmful factors. Salivary immunoglobulins and high-molecular-weight glycoproteins, restrain bacterial metabolism. A group of salivary proteins (such as lysozyme, peroxidase, myeloperoxidase, lactoferrin), together with other salivary components (thiocyanate, chlorine, hydrogen peroxide), are able to interfere with the growth of oral bacteria and fungi. Karoleweska et al. analyzed the salivary flow of 44 children with ALL, finding that the introduction of chemotherapy caused a decrease in salivary secretion rate and S-IgA concentration. Furthermore, patients who developed oral mucositis presented lower myeloperoxidase and peroxidase concentration than those without oral mucositis [47]. Hedge et al. found a deterioration in oral health status and gingival status, with increased dental caries in the group of analyzed children who received chemotherapy for ALL. Moreover, salivary flow rate, salivary pH and total salivary antioxidant levels were lowered in leukemic children when compared with the control group. Total salivary antioxidant levels were increased in leukemic children before the induction of chemotherapy, but their levels were significantly decreased at the end of induction chemotherapy [48].

The inflammation of the oral mucosa observed in pediatric oral mucositis may be scored using the World Health Organization (WHO) system, which grades the oral damage into five stages that occur consecutively and are mechanistically linked (*grade 0*: no change; *grade 1*: soreness/erythema; *grade 2*: erythema, ulcers, can eat solids; *grade 3*: ulcers can eat liquid diet only; *grade 4*: oral alimentation not possible) [49].

The initial injury of the mucosa membranes occurs concurrently with chemotherapy or radiotherapy administration. The type of chemotherapeutic agents, their dosage and the schedule of administration are important factors affecting the severity of the mucosal injury. Chemotherapeutic drugs associated with an important risk of mucositis are alkylating agents (busulfan, cyclophosphamide, procarbazine, thiotepa), anthracyclines (daunorubicin, doxorubicin, and epirubicin), platinum compounds (cisplatin, carboplatin, oxaliplatin), antimetabolite agents (cytosine arabinoside, hydroxyurea, 5-flurouracil, methotrexate, 6-mercaptopurine, and 6-thioguanide), antibiotics (actinomycin D, bleomycin, mitomycin), vinca alkaloids (vinblastin and vincristine) and taxanes (docetaxel), as shown in Table 1. Melphalan, doxorubicin, 5-fluoruracil, methotrexate, and cisplatin etoposide may produce a high stomatotoxic effect, directly causing mucosal breakdown [50].

Correct oral hygiene and a good gingival condition are associated with a lesser incidence of oral mucositis. Basic oral care involves multi-agent combination oral care protocols based on daily oral hygiene with a soft toothbrush and toothpaste, mouthwash with sodium bicarbonate and chlorhexidine. Few therapeutic options are effective for prevention and treatment of oral mucositis and many of them are still being studied. Antioxidant agents (amifostine, glutamine, oral zinc supplement, vitamin E, N-acetyl-cysteine, GC4419), inhibitors of cytokines production (turmeric, clonidine lauriad buccal tablets, pentoxifylline, dusquetide), natural agents (honey, aloe vera gel, chamomile mouthwash, MF5232), oral cryotherapy, and probiotics are currently under investigation for mucositis prevention [51]. Moreover, treatment of pain associated with mucositis should be obtained with opioids, such as morphine. Local mouthwashes with chlorhexidine and 0.2% morphine may allow better pain control than systemic analgesic treatment [52].

Mazhari et al. performed a systematic review and meta-analysis of published studies to investigate the effects of agents and techniques in reducing oral mucositis in children undergoing cancer therapy. They found that Palifermin (at the dosage of 60 micrograms/kg/day from 3 days before to 3 days after chemotherapy) may reduce the incidence, duration and severity of oral mucositis in children receiving cancer therapy [53].

The pathobiology of oral mucositis has been investigated through appropriate animal models, which summarize the human condition. Sonis et al. used an animal model for establishing the stomatotoxicity of chemotherapeutic agents and studying the influence of oral mucosal breakdown on sepsis in myelosuppressed people. They used three intraperitoneal injections of 5-fluorouracil at 5-day intervals and superficial mechanical mucosal irritation for inducing the clinical breakdown of the oral mucosa and ulcerative mucositis in Golden Syrian hamsters. The changes on the oral mucosa of their animal model were similar to those described in human beings [54]. More recently, Shimamura et al. developed a mouse model of oral mucositis induced by chemotherapy, to reduce stomatitis as a side effect of anticancer drugs [55]. Takeouki et al. developed a rat model of oral mucositis, injecting an acetic acid aqueous solution into the buccal mucosa of rats to which a 5-FU solution had been previously administered. In rats, an amount of oral mucosal tissue larger than the mouse model may be obtained for quantitative measurement [56].

In humans, the development of oral mucositis consists of a cascade of events that occur consecutively and are automatically linked.

Systemic chemotherapy induces tissue damage causing reactive oxygen species (ROS) release and DNA damage, leading to cell death of the basal and suprabasal epithelial cells. DNA strand breaks lead to the activation of the apoptotic process, which is regulated by p53 activation and increased caspase 3 and endogenous damage-associated pattern molecules (DAMPs) [57]. The response to this initial damage characterizes the second stage of mucositis development. During this stage the cells of the injured mucosa promote the transcription of several genes involved in the mucositis process. In this molecular scenario, nuclear factor-κB (NF-κB) represents the main transcriptional mediator that modulates over 200 genes associated with proinflammatory cytokines, such as tumor necrosis factor-α (TNF-α), interleukin-6 (IL-6), and interleukin-1β (IL-1β). Pro-inflammatory cytokines colonize oral mucosa, inducing early damage of connective tissue and endothelium, inhibiting tissue oxygenation and promoting epithelial basal cell death [58]. Simultaneously with the activation of other pathways, the primary damage is amplified through a positive feedback loop mechanism. The released TNF-α initiates the activation of the mitogen-activated protein kinase (MAPK) on target cells and sustains NF-κB activity. During this stage, despite the impairment of mucosa and sub-mucosa, patients usually exhibit few symptoms without macroscopic evidence of mucosal injury.

**Figure 1:**
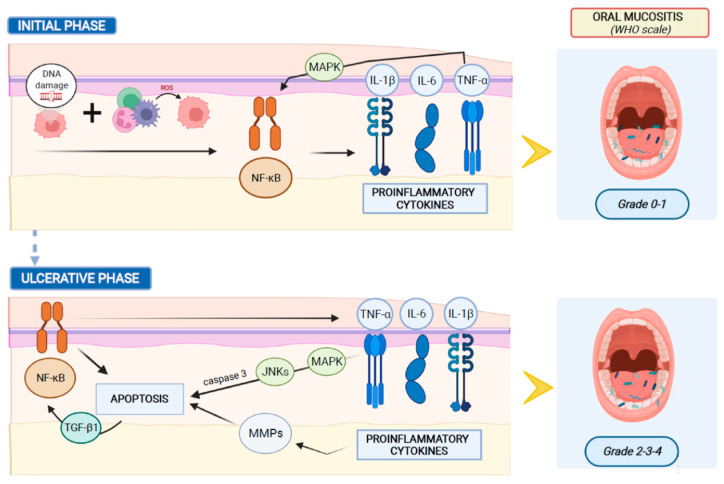
Main mechanisms of oral mucositis pathogenesis.

Afterward, MAPK signaling mediates caspase 3 activation and cell death through the activation of c-Jun N-terminal Kinases (JNKs), which modify Activator Protein 1 (AP1) transcriptional activity. Moreover, the high levels of TNF-α and IL-1β produce an amplification of the pro-apoptotic signal, through the activation of Matrix Metalloproteinase (MMP) activity. Furthermore, the injured keratinocytes release Transforming Growth Factor-beta 1 (TGF-β1), which inhibits the cell cycle, recruits leucocytes and sustains NF-κB activity, improving the damage-mediated signaling [59,60]. Figure 1 shows the main mechanisms of oral mucositis pathogenesis.

Clinical manifestations of mucositis are noticeable at the fourth stage of the inflammation process. During this stage, the mucosa and sub-mucosa are ulcerated, patients complain of pain and oral nutrition is not possible. Furthermore, the breaks in the submucosa promote bacterial translocation, which may explain the involvement of oral microbiota in the pathogenesis of oral mucositis. Symbiotic inhabitant microorganisms of the healthy mucosa invade the damaged submucosa, leading to mononuclear infiltrating cell-mediated inflammation response, which promotes new pro-inflammatory cytokine release, amplifies the expression of pro-apoptotic mediators, and increases the tissue damage [61].

**Figure 2:**
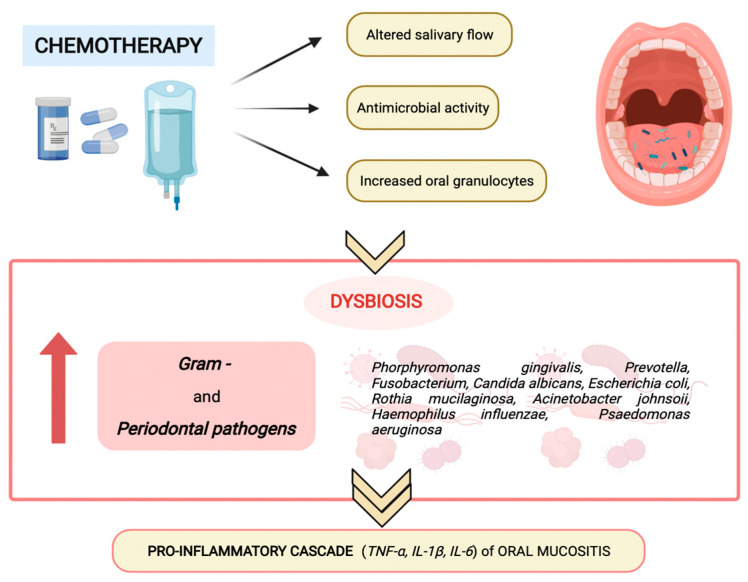
Chemotherapy-induced modification of oral microbiota composition and homeostasis.

The altered salivary production and composition, the augmented oral granulocyte presence and the antimicrobial activity of chemotherapy modify oral microbiota composition and homeostasis, favoring the predominance of Gram-negative anaerobes and periodontal pathogens (as shown in Figure 2), such as *Porphyromonas gingivalis*, *Fusobacterium, Prevotella, Escherichia coli, Haemophilus influenza, Rothia mucilanginosa, Acinetobacter johnsonii, Pseudomonas aeruginosa,* and *Candida albicans*, which promote the proinflammatory cascade of oral mucositis [17].

A limited number of studies have investigated the role of the oral microbiota in the pathogenesis of oral mucositis subsequent to chemotherapy, especially in the pediatric population [52].

Hong et al. investigated the disruption of the oral microbiota in 49 adult patients who received chemotherapy, showing depletion of health-associated commensals (such as *Streptococcus, Actinomyces, Gemella, Granulicatella,* and *Veillonella*) and an enrichment of Gram-negative bacteria and periodontal pathogens (such as *Fusobacterium* and *Prevotella*) [62].

Wang et al. analyzed oral health, caries risk profiles and oral microbiota among 39 pediatric patients with acute lymphoblastic leukemia (ALL) who received primary chemotherapy, comparing them with their healthy counterparts. They found high rates of dental caries, xerostomia, gingivitis and mucositis among ALL children. Comparison of species-level OTUs between ALL patients and healthy children revealed that the ALL patients group exhibited substantial dysbiosis of the oral microbiota, characterized by lower richness and less diversity than the healthy controls [63].

Ye et al. conducted a preliminary study among 37 pediatric patients who received chemotherapy compared with a control group. They discovered that those patients who developed oral mucositis presented a more heterogeneous bacterial community at diagnosis and then developed a more pronounced change of bacterial composition, with higher microbial diversity after the initiation of chemotherapy. No mucositis group seemed to be less modified by chemotherapy, as an expression of higher microbial stability [64,65].

## 4. Conclusions

Among pediatric patients with cancer, oral mucositis may occur as a common and painful side effect subsequent to chemotherapy, even when conventional dosing schemes are adopted. The pathobiology of oral mucositis is complex and multifaceted, giving several opportunities for personalized interventions.

During cancer treatment, the oral microbiota undergoes a dysbiosis, characterized by a substantially lower richness and less diversity of species, which seems to support the pro-inflammatory cascade observed in the pathogenesis of oral mucositis.

Oral mucositis represents a common side effect among children who undergo chemotherapy, with a negative effect both on the quality of life and on the adherence to the treatment. We recommend further studies on the modification of oral microbiota among children with cancer who receive chemotherapy. The role of the oral microbiota seems to be crucial and should be investigated in the pathogenesis of oral mucositis, for discovering preventive and protective factors against this treatment-associated side effect.

## Figures and Tables

**Table 1:** Stomatotoxic effect of principal chemotherapeutic drugs.

	High Stomatotoxic Effect (Directly Mucosal Breakdown)	Moderate Stomatotoxic Effect
*Alkylating agents*	Melphalan	Busulfan, cyclophosphamide, procarbazine, thiotepa, mechlorethamine
*Anthracyclines*	Doxorubicin	Daunorubicin, epirubicin
*Antimetabolite agents*	Cytarabine, 5-fluoruracil, methotrexate	Hydroxyurea, 6-mercaptopurine, 6-thioguanine
*Platinum compounds*	Cisplatin	Carboplatin, oxaliplatin
*Antibiotics*	-	Actinomycin D, bleomycin, mytomicin
*Vinca alkaloids*	-	Vinblastin, vincristine, vinorelbine
*Taxanes*	-	Docetaxel, paclitaxel
*Others*	Etoposide	Lomustine

## Data Availability

Not applicable.
